# A Critical Regulatory Role for Macrophage Migration Inhibitory Factor in Hyperoxia-Induced Injury in the Developing Murine Lung

**DOI:** 10.1371/journal.pone.0060560

**Published:** 2013-04-29

**Authors:** Huanxing Sun, Rayman Choo-Wing, Angara Sureshbabu, Juan Fan, Lin Leng, Shuang Yu, Dianhua Jiang, Paul Noble, Robert J. Homer, Richard Bucala, Vineet Bhandari

**Affiliations:** 1 Department of Pediatrics, Yale University, New Haven, Connecticut, United States of America; 2 Department of Medicine, Yale University, New Haven, Connecticut, United States of America; 3 Department of Medicine, Duke University School of Medicine, Durham, North Carolina, United States of America; 4 Department of Pathology, Yale University, New Haven, Connecticut, United States of America; University of Washington, United States of America

## Abstract

**Background:**

The role and mechanism of action of MIF in hyperoxia-induced acute lung injury (HALI) in the newborn lung are not known. We hypothesized that MIF is a critical regulatory molecule in HALI in the developing lung.

**Methodology:**

We studied newborn wild type (WT), MIF knockout (MIFKO), and MIF lung transgenic (MIFTG) mice in room air and hyperoxia exposure for 7 postnatal (PN) days. Lung morphometry was performed and mRNA and protein expression of vascular mediators were analyzed.

**Results:**

MIF mRNA and protein expression were significantly increased in WT lungs at PN7 of hyperoxia exposure. The pattern of expression of Angiopoietin 2 protein (in MIFKO>WT>MIFTG) was similar to the mortality pattern (MIFKO>WT>MIFTG) in hyperoxia at PN7. In room air, MIFKO and MIFTG had modest but significant increases in chord length, compared to WT. This was associated with decreased expression of Angiopoietin 1 and Tie 2 proteins in the MIFKO and MIFTG, as compared to the WT control lungs in room air. However, on hyperoxia exposure, while the chord length was increased from their respective room air controls, there were no differences between the 3 genotypes.

**Conclusion:**

These data point to the potential roles of Angiopoietins 1, 2 and their receptor Tie2 in the MIF-regulated response in room air and upon hyperoxia exposure in the neonatal lung.

## Introduction

Hyperoxia-induced lung injury is characterized by an influx of inflammatory cells, increased pulmonary permeability, and endothelial and epithelial cell injury/death [1], [2]. A variety of molecular mediators, including cytokines have been implicated as having a critical role in these processes [1], [2]. There are significant differences in the response to HALI in the developing and mature lungs, and it is important to identify and understand such unique responses in order to assess potential therapeutic approaches to HALI in such circumstances [1], [3]. We have recently reported that loss of macrophage migration inhibitory factor (MIF) in the mouse leads to impaired lung maturation, akin to a respiratory distress syndrome (RDS) in premature newborns, and increased mortality [4]. Neonates with RDS are frequently exposed to high concentrations of oxygen for a prolonged duration as part of their therapeutic regimen. Surprisingly, we found no information about MIF expression on exposure to hyperoxia, or the role, if any, in HALI in the developing lung.

Hence, we hypothesized that MIF is a critical regulatory molecule in HALI in the newborn lung. To address this hypothesis, we studied newborn (NB) mice genetically deficient in MIF (MIF null mutant or knock out: MIFKO). We generated a novel lung-targeted MIF overexpressing TG (MIFTG) mouse and examined the phenotype. These genetically defined mouse strains were further subjected to a HALI model, with sustained exposure to hyperoxia for 7 postnatal (PN) days. Given previous reports by us [4] and others [5]–[13] about VEGF being involved in MIF signaling and the potential role of VEGF in alveolar simplification in the developing lung, we also evaluated NB MIFKO mice crossbred with the vascular endothelial factor (VEGF) overexpressing TG mice.

Our goal was to study alterations in pulmonary phenotype and the expression of vascular mediators in the varied mouse models. Specifically, we evaluated lung morphometry and the expression of vascular endothelial growth factor (VEGF), its receptors (R1–3), angiopoietin (Ang), and its receptor (Tie2) in the lung.

## Materials and Methods

### Animals

MIFKO mice were backcrossed for this study into the C57BL/6 genetic background (generation N10) [4]. A transgenic mouse overexpressing MIF in Type II lung alveolar cells, which produce MIF under basal conditions [14], was created by a previously established methodology [15]. Briefly, a 0.4 Kb DNA fragment containing the complete mouse MIF cDNA [16] was inserted into the Xbal/Spel site of an expression plasmid under the control of the rat CC10, which is a Type II epithelial-cell and conducting airway-cell specific promoter in the developing lung [17]. Plasmid transfection into Bruce4 ES cells permitted immediate establishment of founder mice in the pure C57BL/6 background. Immunohistochemistry analysis at 6 weeks confirmed MIF overexpression in alveolar epithelium and quantitative, real-time PCR analysis of MIF mRNA in lung tissue and MIF protein levels in bronchoalveolar lavage fluid revealed a 2.4 and 4.3 fold increase in MIF mRNA transcripts and MIF protein, respectively (**figure S1**).

Lung-targeted and regulatable VEGF TG mice overexpressing VEGF_165_, a kind gift from Jack Elias, MD, were generated and characterized as previously described [18]–[20], and were cross-bred with MIF KO mice. The VEGF TG mice express VEGF_165_ in the lung with maternal exposure to doxycycline (dox) in the drinking water, leading to transmammary activation in the TG (+) pups [20]. Maternal exposure to dox was performed from PN1 to PN7.

All animal work was approved by the Institutional Animal Care and Use Committee at the Yale University School of Medicine.

### Oxygen exposure

For the NB animals, exposure to hyperoxia (along with their mothers) was performed by placing mice in cages in an airtight Plexiglass chamber (55×40×50 cm), as described previously [20], [21]. For the NB survival experiments, exposure to 100% oxygen was initiated on PN1 and continued until PN7. Two lactating dams were used and alternated in hyperoxia and room air (RA) every 24 h. The litter size was limited to 10–12 pups per dam to control for the effects of litter size on nutrition and growth.

Throughout the experiment, mice were given free access to food and water, and oxygen levels were continually monitored. The inside of the chamber was maintained at atmospheric pressure and mice were exposed to a 12 hr light-dark cycle.

### Analysis of mRNA

RNA was isolated from frozen lungs using TRIzol Reagent (Invitrogen Corporation, Carlsbad, CA) and treated by DNase. RNA samples were then purified by RNeasy kit (Qiagen Sciences, Maryland, USA) according to the manufacturer's instructions. RNA samples were subjected to real time PCR and semi-quantitative RT-PCR. The primers used for real time PCR:

MIF-F: 5′-CCA GAA CCG CAA CTA CAG TAA -3′

MIF-R: 5′ -CCG GTG GAT AAA CAC AGA AC -3′

The primers used for semi-quantitative RT-PCR are as follows:

β-Actin-F, 5′-GTGGGCCGCTCTAGGCACCA -3′

β-Actin-R, 5′-TGGCCTTAGGGTTCAGGGGG -3′

VEGF-A-F, 5′- GACCCTGGCTTTACTGCTGTA -3′

VEGF-A-R, 5′- GTGAGGTTTGATCCGCATGAT -3′

VEGF-C-F, 5′- AACGTGTCCAAGAAATCAGCC -3′

VEGF-C-R, 5′- AGTCCTCTCCCGCAGTAATCC -3′

VEGF-R1-F, 5- CACCACAATCACTCCAAAGAAA -3′

VEGF-R1-R, 5- CACCAATGTGCTAACCGTCTTA -3′

VEGF-R2-F, 5- ATTGTAAACCGGGATGTGAAAC -3′

VEGF-R2-R, 5- TACTTCACAGGGATTCGGACTT -3′

VEGF-R3-F, 5- GCTGTTGGTTGGAGAGAAGC -3′

VEGF-R3-R, 5- TGCTGGAGAGTTCTGTGTGG -3′

Ang1-F, 5′- AGGCTTGGTTTCTCGTCAGA -3′

Ang1-R, 5′- TCTGCACAGTCTCGAAATGG -3′

Ang2-F, 5′- GAACCAGACAGCAGCACAAA -3′

Ang2-R, 5′- AGTTGGGGAAGGTCAGTGTG -3′

Tie2-F, 5′- GGACAGTGCTCCAACCAAAT -3′

Tie2-R, 5′- TTGGCAGGAGACTGAGACCT -3′

mRNA band densities were measured by densitometry using NIH image J and expressed in Arbitrary Densitometric Units (ADU), as previously described [21].

### Histology

Lung tissues obtained from the NB mice from the RA experiments at PN7 were subjected to a standard protocol for lung inflation (25 cm) and fixed overnight in 10% buffered formalin [22]. After washing in fresh PBS, fixed tissues were dehydrated, cleared, and embedded in paraffin by routine methods. Sections (5 µm) were collected on Superfrost Plus positively charged microscope slides (Fisher Scientific Co., Houston, Texas, USA), deparaffinized, and stained with hematoxylin & eosin, as described previously [20].

### Lung Morphometry

Alveolar size was estimated from the mean chord length of the airspace, as described previously [20]. Chord length increases with alveolar enlargement. Alveolar septal wall thickness was estimated using Image J software, adapting the method described previously for bone trabecular thickness, for lung [23].

### Bronchoalveolar lavage (BAL) fluid total and differential cell counts and IL-6 levels

BAL fluid was obtained and total and differential cell counts enumerated, as previously reported [24], [25]. IL-6 levels were measured using ELISA (R&D Systems, Minneapolis, MN), as per manufacturer's instructions.

### Quantitative measurements of Pulmonary artery hypertension (PAH)-induced right ventricular hypertrophy (RVH)

We assessed quantitative measurements of PAH-induced RVH by RV/left ventricle (LV) and RV/(LV + interventricular septum or IVS) ratios, using the methodology as described previously [26].

### Western Blotting

We detected MIF, Ang1, Ang2, Tie2, VEGF-A, and VEGFR1 protein with β-actin as control from lung lysates using Western analysis, as described previously [20], [25]. Anti-MIF specific antibody was purchased from Abcam (Cambridge, MA). Ang1 and Ang2 antibodies were purchased from Millipore (Billerica, MA). VEGF-A and β-actin antibodies were purchased from Santa Cruz Biotechnology, Santa Cruz, CA. VEGFR1 and Tie2 antibodies were obtained from Cell Signaling, Danvers, MA.

### Statistical Analyses

Values are expressed as mean ± SEM. Groups were compared with the Student's two-tailed unpaired *t* test or the logrank test (for the survival analysis), using GraphPad Prism 3.0 (GraphPad Software, Inc., San Diego, CA), as appropriate. A *P*<0.05 was considered statistically significant.

## Results

### Effect of hyperoxia on MIF expression in NB WT, MIFKO and MIFTG mice lungs

**Figure 1:**
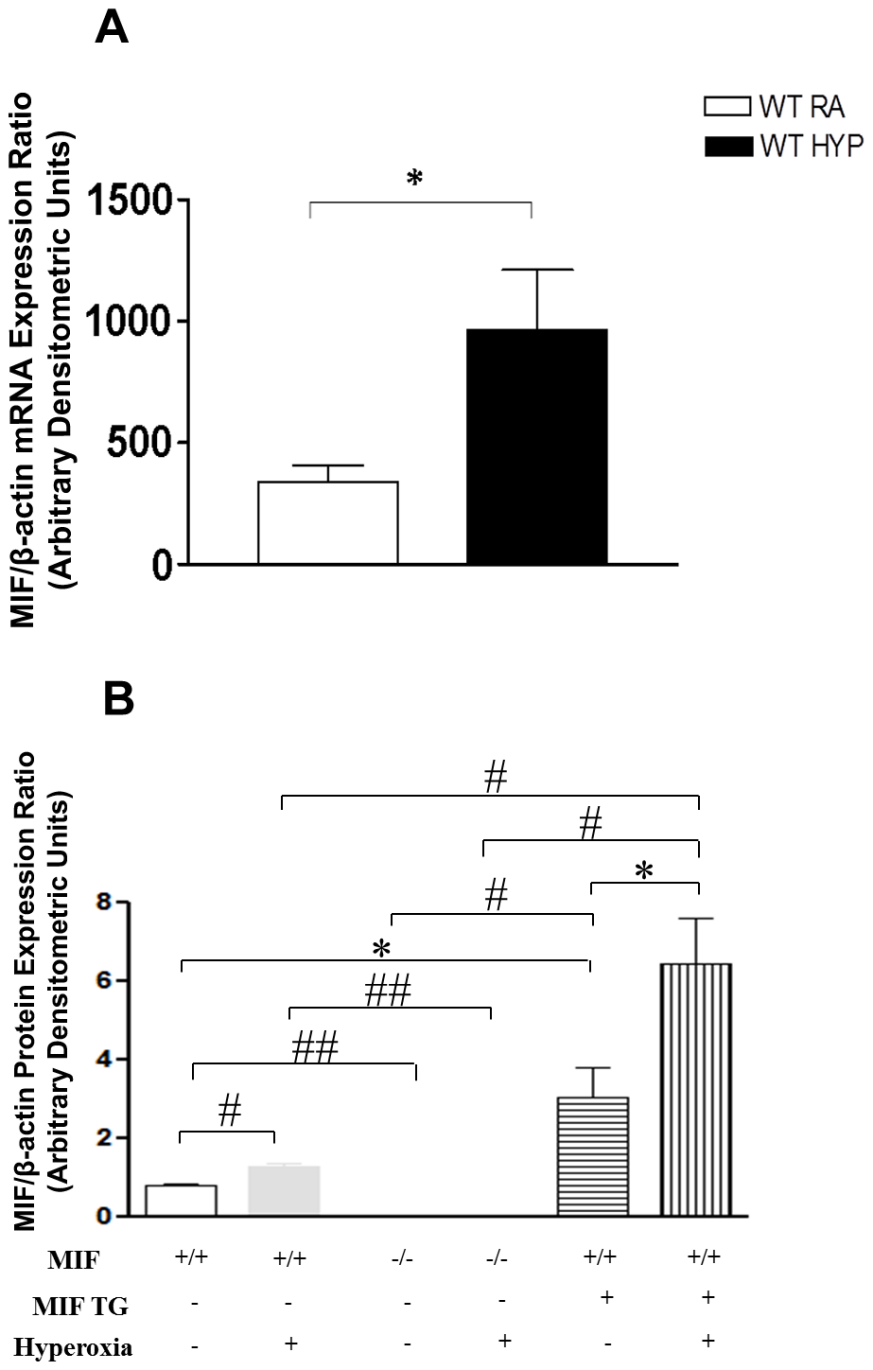
Effect of hyperoxia on MIF expression in NB WT, MIF KO and MIF TG mice lungs at PN7. (**A**) Quantitative real-time PCR of MIF mRNA expression in lungs of NB mice exposed to hyperoxia (as described in “Methods” section) at PN7. Control lungs were exposed to room air for 7 days. The figure is representative of n = 3–4 mice per group. (**B**) MIF protein expression data from WT, NB MIF KO and MIF TG mice lungs at PN7 in RA and exposed to 100% O2 at PN7. The figure is representative of n = 4 mice per group. NB: newborn; WT: wild type; MIF KO: macrophage migration inhibitory factor knock out; MIF TG: macrophage migration inhibitory factor (over-expressing) transgenic; β: beta; HYP: hyperoxia; PN: post natal; RA: room air. *P≤0.05; #*P*≤0.01; ##*P*<0.0001.

Using the hyperoxia-exposed NB WT mouse model, we noted significantly increased expression of lung MIF mRNA (**figure 1A**). MIF protein expression data from NB WT, MIFKO and MIFTG mice lungs at PN7 in RA and after exposure to 100% O_2_ at PN7 has been shown in **figure 1B**.

### Effect of hyperoxia on survival in NB WT, MIFKO and MIFTG mice

**Figure 2:**
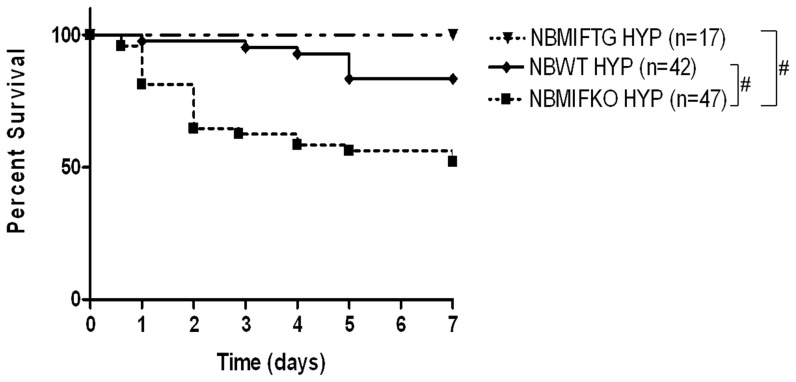
Effect of hyperoxia on survival in NB MIF KO and MIF TG mice at PN7. NB MIF KO, MIF TG and WT control mice were exposed to 100% O_2_ and survival was assessed. The noted values represent the number of animals in each group. NB: newborn; WT: wild type; MIF KO: macrophage migration inhibitory factor knock out; MIF TG: macrophage migration inhibitory factor (over-expressing) transgenic; HYP: hyperoxia. #*P*≤0.01.

There was no statistically significant difference in survival in RA (up to 7 days) of MIFKO mice (MIFKO; n = 24; survival = 82.6%) or lung-targeted, over-expressing MIFTG mice (MIFTG; n = 12; survival = 91.7%) when compared to WT mice (n = 26; survival = 92.3%). However, when we evaluated the survival of MIFKO mice and MIFTG mice after hyperoxia for 7 days, there was a significant decrease in survival of the MIFKO mice when compared to WT controls at PN7 (p≤0.01) (**figure 2**). Notably, the MIFTG mice had significantly increased survival compared to the MIFKO mice at PN7 (p≤0.01) (**figure 2**). Similarly, there was a trend towards increased survival of MIFTG, compared to WT mice (p = 0.08) (**figure 2**).

### Effect of hyperoxia on lung architecture in NB MIFKO and MIFTG mice

**Figure 3:**
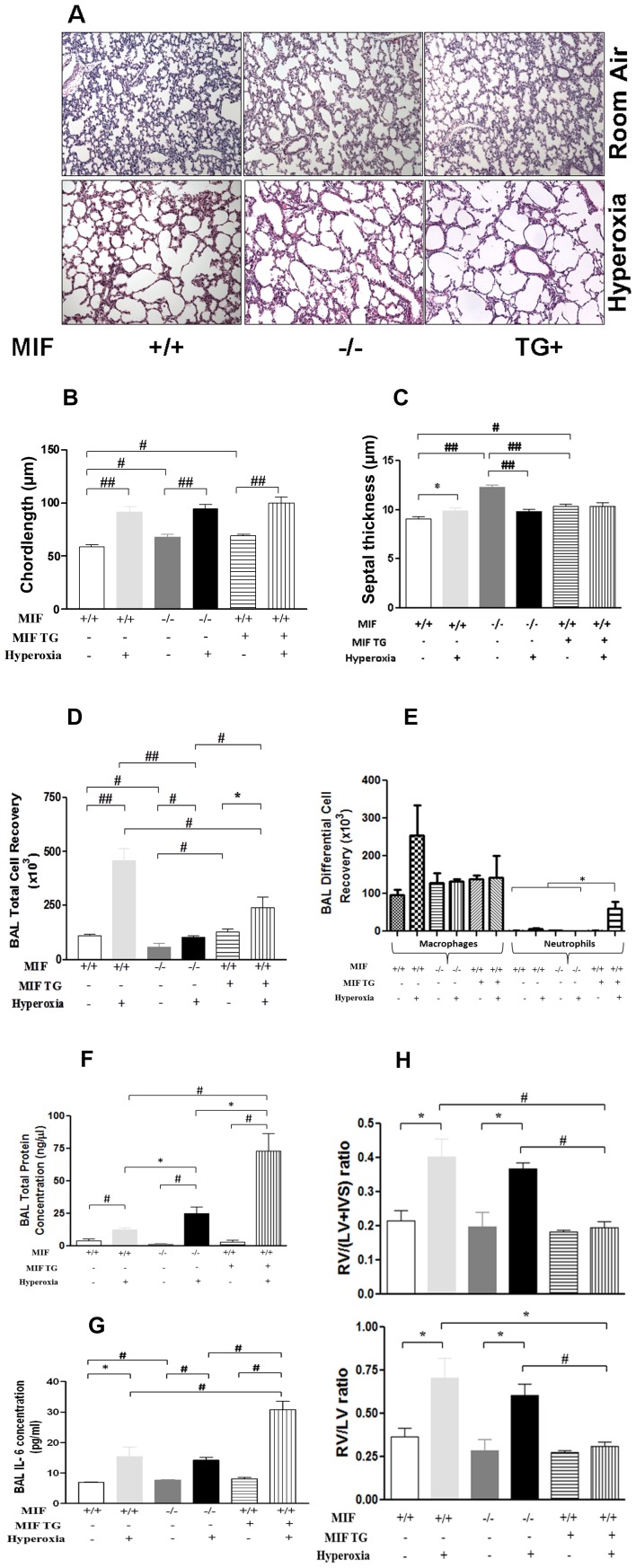
Effect of hyperoxia on lung architecture, BAL total and differential cell counts, BAL total protein concentrations and IL-6 levels in NB MIF KO and MIF TG mice at PN7. Representative photomicrographs of lung histology (H&E stain) of NB MIF KO, MIF TG and WT control mice exposed to RA or survived 100% O_2_ at PN7 (3A). The figures are illustrative of a minimum of 8 animals in each group. Alveolar size, as measured by chord length and septal thickness, confirmed features noted on lung histology (3B and 3C). Each bar represents the mean ± SEM of a minimum of 5 animals. BAL total and differential cell recovery of NB MIF KO, MIF TG and WT control mice exposed to RA or survived 100% O_2_ at PN7 (3D and 3E). Each bar represents the mean ± SEM of a minimum of 12 animals. BAL total protein levels of NB MIF KO, MIF TG and WT control mice exposed to RA or survived 100% O_2_ at PN7 (3F). Each bar represents the mean ± SEM of a minimum of 4 animals. BAL IL-6 levels of NB MIF KO, MIF TG and WT control mice exposed to RA or survived 100% O_2_ at PN7 (3G). Quantitative measurements of RV hypertrophy (3H).^§^Each bar represents the mean ± SEM of a minimum of 3 animals. MIF +/+: wild type; MIF −/−: macrophage migration inhibitory factor knock out; MIF TG +: macrophage migration inhibitory factor (over-expressing) transgenic; BAL: bronchoalveolar lavage; IL-6: interleukin-6; RV: right ventricle; LV: left ventricle; IVS: interventricular septum. ^§^Note: MIF KO assessments were done at PN6, due to their increased mortality in hyperoxia at PN7. ##*P*<0.0001, #*P*≤0.01, *P<0.05.

Surprisingly, both the MIFKO and MIFTG mouse lungs exhibited simplified alveoli with a reduction in secondary crests when compared to the WT control mice in RA (**figure 3A**). After hyperoxia exposure from PN1-7, there was a significant alveolar simplification in NB WT, MIFKO and MIFTG mice when compared to their respective RA controls (**figure 3A**) However, the alveolar simplification appeared to be of a similar degree in the NB WT, MIFKO and MIFTG mice lungs exposed to hyperoxia till PN7(**figure 3A**). These results were confirmed by lung morphometry on chord length measurements (**figure 3B**). Septal thickness was higher in NB WT mice exposed to hyperoxia, compared to RA (**figure 3C**). NB MIFKO mice lungs had the highest septal thickness, which decreased on hyperoxia exposure, but with values similar to that of NB WT in hyperoxia (**figure 3C**). NB MIFTG mice lungs in RA or hyperoxia-exposed also had similar values to that of NB WT in hyperoxia (**figure 3C**).

Taken together, these data suggest that either a lack or an excess of MIF in the developing lung in RA results in significant alterations in pulmonary architecture.

### Effect of hyperoxia on BAL total cell counts, total protein and IL-6 levels in NB MIFKO and MIFTG mice

To gain insight into potential mechanisms underlying alveolar simplification and the increased survival of the NB MIFTG mice on hyperoxia exposure, we evaluated cell counts in BAL fluid obtained from MIFKO, MIFTG, and WT mice subjected to RA or hyperoxia. In RA, MIFKO animals had the lowest cell numbers among the experimental groups (**figure 3D**). As anticipated, exposure to hyperoxia resulted in a significant increase in the total BAL cell count at PN7 in WT animals (**figure 3D**). A similar pattern was noted in the lungs of MIFKO and MIFTG mice that survived exposure to hyperoxia (**figure 3D**). On exposure to hyperoxia, WT animals had the most robust increase in BAL total cell counts, and it was significantly higher than the cell counts observed in the MIFTG, which, in turn, was higher than in MIFKO mice (**figure 3D**). In the differential cell counts, the majority of the cells in RA or hyperoxia were macrophages. Interestingly, there was a marked increase in neutrophils in the MIFTG mice in hyperoxia (**figure 3E**).

BAL total protein concentrations were significantly increased in the hyperoxia-exposed animals (**figure 3F**), compared to their respective RA controls. The BAL total protein was significantly higher in the MIFKO vs. the WT mice exposed to hyperoxia. However, the highest increase of BAL total protein concentration was noted in the MIFTG mice.

BAL IL-6 levels were significantly increased in the hyperoxia-exposed animals (**figure 3G**). The highest increase of BAL IL-6 levels was noted in the MIFTG mice, with similar concentrations noted in the WT and KO mice exposed to hyperoxia.

Measurements of PAH-induced RVH revealed that these values were significantly increased in the WT and MIFKO mice exposed to hyperoxia, but significantly decreased in the MIFTG mice exposed to hyperoxia (**figure 3H**).

This suggests that enhanced inflammation, as assessed by BAL fluid cell counts, total protein concentration and IL-6 levels, could account for the differences in lung architecture after hyperoxia exposure when compared to RA. Interestingly, the MIFTG mice exposed to hyperoxia appeared to be protected against PAH-induced RVH. However, an explanation for the alveolar simplification noted in RA in the MIFKO and MIFTG mice, and the increased survival of the NB MIFTG mice on hyperoxia exposure was still required.

### Effect of hyperoxia on mRNA and protein expression of vascular growth factors and their receptors in MIFKO and MIFTG mice

**Figure 4:**
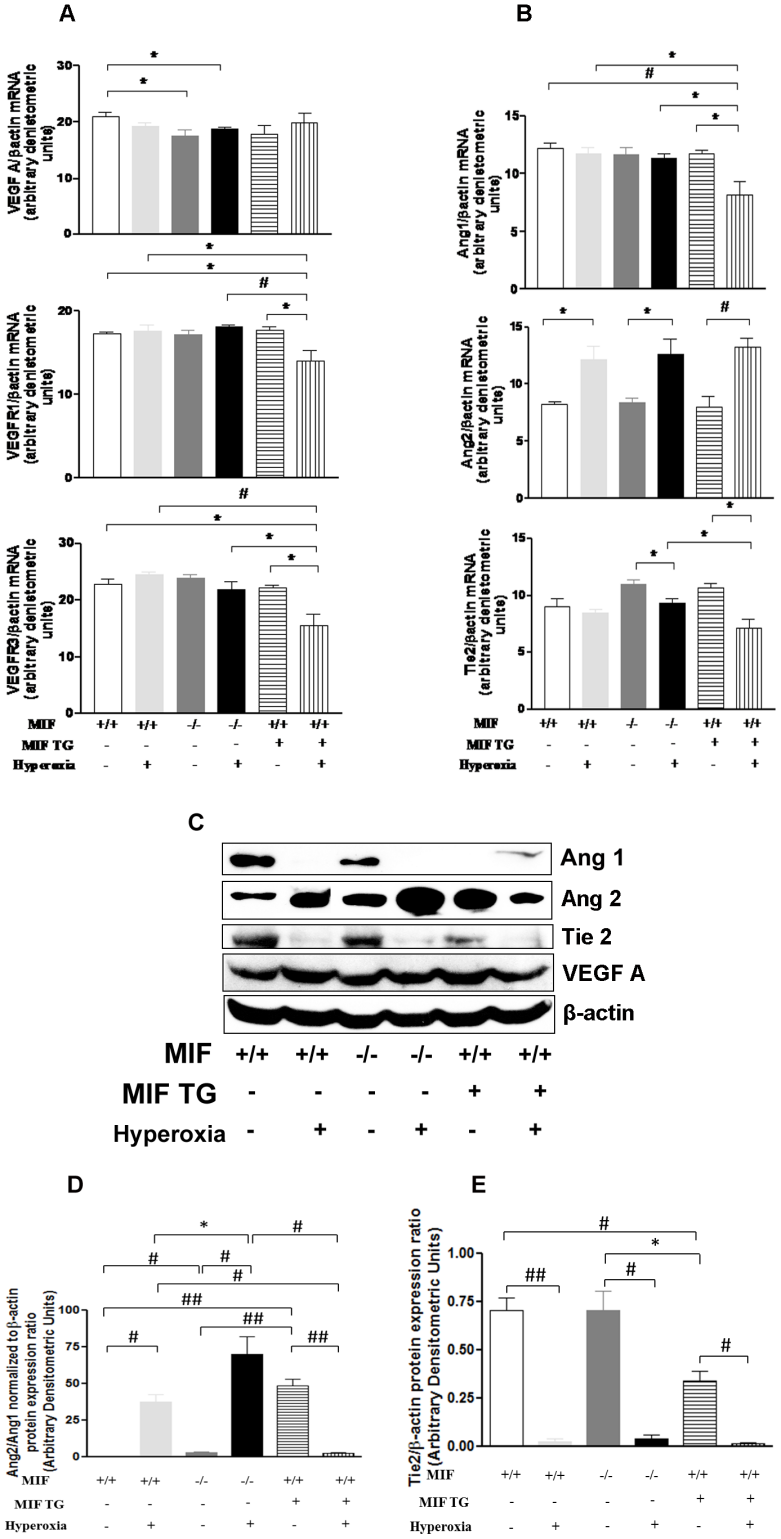
Effect of hyperoxia on RNA expression of vascular mediators and their receptors in NB MIF KO and MIF TG mice at PN7. The ratios of mRNA of VEGF -A, and the receptors -R1 and -R3 (4A) as well as Ang1 and Ang2 and their receptor Tie2 (4B) with β-actin were quantified by densitometery. Ang1, Ang2, Tie2, and VEGF-A proteins, with β-actin as controls, were detected by western blotting (4C). The densitometric values of Ang2/Ang1 ratio and Tie2 protein expression are noted in 4D and 4E, respectively. The noted values represent assessments in a minimum of 4 animals in each group. MIF +/+: wild type; MIF −/−: macrophage migration inhibitory factor knock out; MIF TG +: macrophage migration inhibitory factor (over-expressing) transgenic; VEGF: vascular endothelial growth factor; R: receptor; Ang: angiopoietin; Tie2: Tyrosine kinase with Ig and EGF homology domain 2 (receptor for Ang1 and 2); β-actin: beta actin. ##*P*<0.0001, #*P*≤0.01, *P<0.05.

Since alveolar simplification is associated with impaired vasculogenesis [27], we next studied the mRNA expression of vascular endothelial growth factors (VEGF) -A and –C, and their receptors -R1, -R2 and -R3. In addition, we measured Angiopoietin (Ang)-1 and 2, and their common receptor, Tie2 (**figures 4A**–**B**). As shown in **figure 4A**, there was a significant reduction in mRNA expression of VEGF-A in the MIF KO mice lungs in RA and after hyperoxia when compared to the WT mice in RA at PN7. No differences were observed in the expression levels of VEGF-C and VEGF-R2 mRNA (data not shown). Interestingly, MIFTG mice exposed to hyperoxia had consistently decreased expression of VEGF -R1 and -R3 mRNA, compared to WT or MIFTG mice in RA, as well as WT or MIFKO in hyperoxia (**figure 4A**). A similar pattern of decreased expression of Ang1 was also observed in MIF TG mice lungs exposed to hyperoxia when compared to WT or MIFKO mice (**figure 4B**). However, the expression of Ang2 mRNA was significantly increased in the hyperoxia-exposed mouse lungs when compared to their respective RA controls (**figure 4B**). Finally, Tie2 mRNA expression was decreased in the MIFKO and MIFTG mouse lungs after hyperoxia when compared to their respective RA controls (**figure 4B**) and MIFTG mouse lungs showed a significant decrease in the expression of Tie2 after hyperoxia when compared to hyperoxia-exposed MIFKO mouse lungs (**figure 4B**).

We sought to confirm the modest differences in mRNA at the protein level (**figure 4C**). VEGF-A protein expression appeared to be similar in the WT, MIFKO, and MIFTG mice. Ang1 and Tie2 expression were lower in the MIFKO and MIFTG lungs, compared to WT. Ang1 and Tie2 protein expression decreased further in all the mouse strains after hyperoxia exposure. Although Ang2 protein expression decreased in the MIFTG group after hyperoxia exposure, it was increased in the WT and MIFKO groups, which contrasted with the results obtained by RNA expression. This pattern may reflect either impaired release of the protein or its uptake and retention by hyperoxia-exposed cells in WT and MIFKO groups. The protein expression has been quantified in figures **4D and 4E**.

In summary, the lack of MIF in the murine developing lung appears not to influence VEGF-A protein expression while a lack or excess of MIF is associated with a significant reduction in Ang1 and Tie2 proteins. Exposure to hyperoxia led to a further decrease in Ang1 protein, and an increase in Ang2 protein, except in the case of excess MIF expressed by MIFTG lungs, where a decrease in Ang2 protein was noted (**4C and 4D**).

### Effect of lung-targeted VEGF overexpression in MIF KO mice lungs

**Figure 5:**
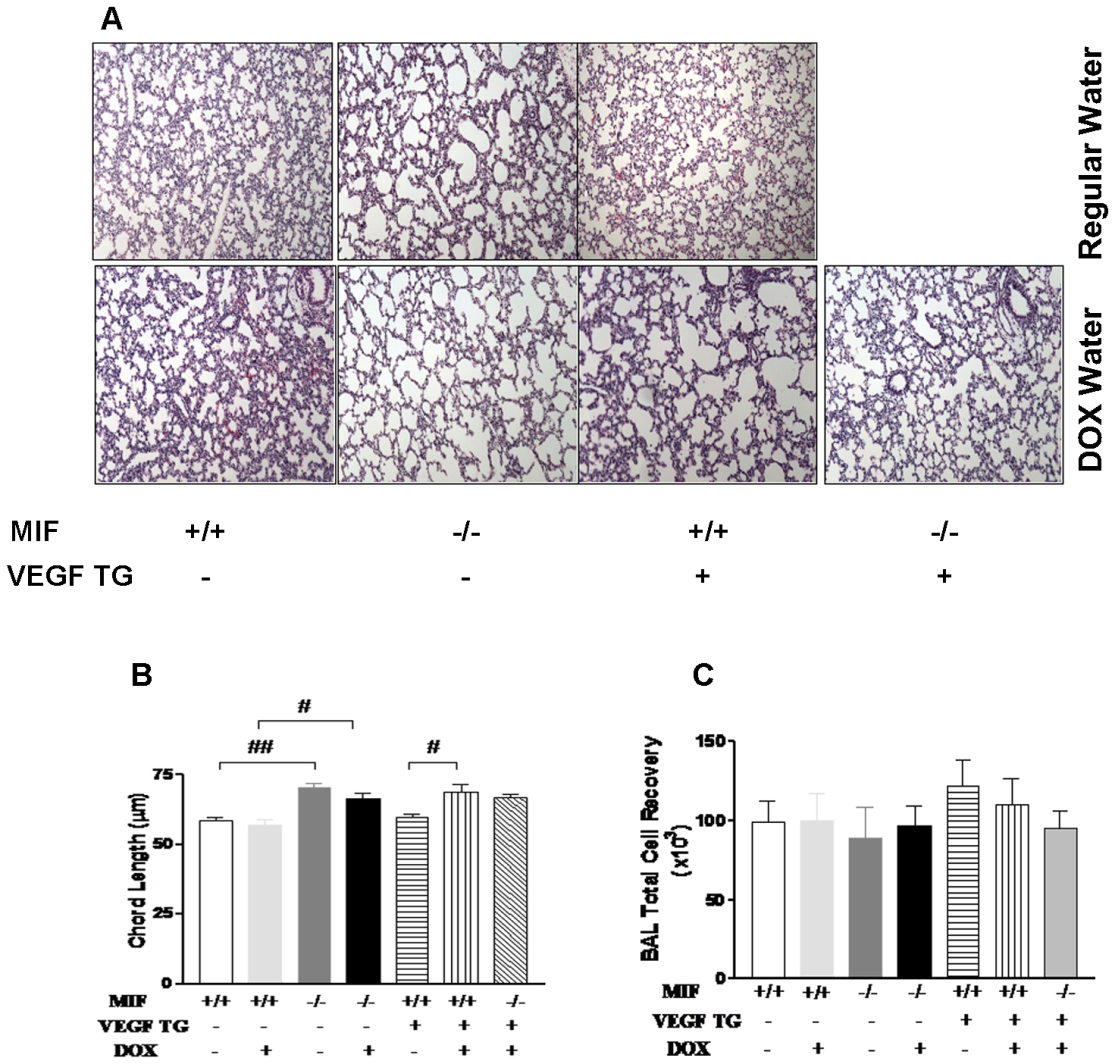
Effect of lung-targeted VEGF overexpression in NB MIF KO mouse lungs. Representative photomicrographs of lung histology (H&E stain) of NB MIF KO, MIF KO VEGF TG and WT litter-mate controls, on regular or dox (to activate VEGF165 in the VEGF TG mice) water mice in RA at PN7 (**5A**). The figures are illustrative of a minimum of 4 animals in each group. Alveolar size, as measured by chord length, confirmed features noted on lung histology (**5B**). Each bar represents the mean ± SEM of a minimum of three animals. BAL total cell count of NB MIF KO, MIF KO VEGF TG and WT litter-mate controls, on regular or dox (to activate VEGF165 in the VEGF TG mice) water mice in RA at PN7 (**5C**). Each bar represents the mean ± SEM of a minimum of three animals. VEGF: vascular endothelial growth factor; MIF +/+: wild type; MIF −/−: macrophage migration inhibitory factor knock out; VEGF TG+: vascular endothelial growth factor (over-expressing) transgenic; dox: doxycycline; BAL: bronchoalveolar lavage. ##*P*<0.0001, #*P*≤0.01.

Our data did not show a difference in VEGF-A protein expression in the WT and MIFKO mice in RA (**figure 4C**). However, earlier reports have suggested VEGF involvement in MIF signaling [7], [9], [12], [28] and alveolar simplification in the developing lung [4]. To test if enhanced VEGF signaling downstream of MIF could impact on lung architecture in the NB lung, we studied an externally-regulatable, lung-targeted VEGF_165_ TG mouse cross-bred with the MIFKO mouse in RA. In our earlier study of VEGFTG mice in RA, we had noted thinning of the mesenchyme and larger alveoli that, in conjunction with increased expression and production of surfactant, suggested an enhancement of lung maturation [20]. Given the impaired lung maturation of the MIF KO mice [4], we wondered if provision of “exogenous” VEGF could enhance lung maturation in such a scenario. An examination of VEGF TG × MIF KO mice in RA revealed no significant recovery of the pulmonary phenotype and no difference in the BAL total cell counts (**figures 5A**–**C**) suggesting that VEGF and MIF function through non-overlapping pathways of lung development. This result was confirmatory of our data noting no effect of VEGF-A protein expression in the MIFKO, compared to WT (**figure 4C**).

## Discussion

We have reported earlier that loss of MIF led to lung immaturity, a condition akin to RDS in premature NB [4]. Recently, in a preterm lamb model, it was suggested that MIF might be an important maturational and protective factor for NB lungs [29]. Since RDS in NB infants is almost always managed with high concentrations of supplemental oxygen, the present studies were undertaken to better define MIF's role in the developing lung in RA and in response to hyperoxia. We employed both genetic loss- and gain-of-function strategies with MIFKO and lung MIFTG mice, respectively.

Sustained and prolonged exposure to hyperoxia to the WT NB mouse lung in the saccular (PN1-4) and alveolar (>PN5) stages of development led to a significantly increased expression of MIF – both at the mRNA and protein level. This could be a damaging or a protective response. To test this, we exposed NB MIFKO, and a novel lung-targeted overexpressing MIFTG mouse, along with WT controls to hyperoxia. In accord with loss of MIF being harmful, there was significantly increased mortality in the MIFKO mice, compared to WT (and MIFTG) mice. Importantly, MIFTG mice had the best survival of all 3 groups. This would suggest that enhanced expression of MIF in the WT mouse on exposure to hyperoxia was a protective response. It is interesting to speculate that given the fact that NB mice are inherently resistant to HALI, vis-à-vis their adults counterparts (of the same strain) [1]–[3], MIF could be responsible, in part, for this enhanced survival response to HALI.

Next, we sought to establish the reason for the increased survival of the MIFTG mice in hyperoxia. The BAL cell count, though lower in the MIFTG vs. WT, was significantly higher than that of the MIFKO mice. Hence, while we cannot rule out a different inflammatory response in the lung parenchyma, we believe the degree of inflammation (as assessed by BAL cell counts) did not sufficiently explain the protective effects in hyperoxia. If the BAL total protein concentrations is taken as a surrogate marker for alveolar-capillary integrity [30], [31], the increased amounts in the MIFKO mice vs. WT in hyperoxia would suggest that there may be more vascular leak/pulmonary edema in the MIFKO mice; thus, providing 1 possible explanation for their increased mortality. However, the highest BAL total protein concentration was noted in in the MIFTG mice lungs in hyperoxia, which had the best survival among the 3 groups. This would suggest that despite significant vascular leak/pulmonary edema, increased release/secretion of MIF itself and/or some other factor/protein was possibly contributing to the protective response in hyperoxia [32], [33].

Hence, we also measured BAL IL-6 levels, and to our surprise, the highest concentrations were noted in the MIFTG mice exposed to hyperoxia. The marked increase in neutrophils in the MIFTG mice in hyperoxia appears to correlate with the increased IL-6 levels in the same mice. While traditionally, IL-6 is considered “pro-inflammatory”, high levels of IL-6 in a lung-targeted mouse model of HALI was protective [21]. However, the same IL-6 TG mouse model in the NB period was exquisitely sensitive to HALI, and had significantly increased mortality [21]. It is possible that there is a threshold effect of IL-6 levels in the developing lungs exposed to hyperoxia in terms of it being damaging or protective, but additional research would be needed to sort this out.

The MIFKO mice appeared to have a similar degree of increased PAH-induced RVH as the WT mice exposed to hyperoxia; the latter response has also been noted in neonatal rats [26]. However, the MIFTG mice exposed to hyperoxia appeared to be protected against PAH-induced RVH. These data are supportive of their increased survival on hyperoxia exposure. Further research will be required to understand the mechanism of this differential response.

Since angiogenic agents have been implicated in the beneficial response to HALI in developing lung models [1], [20], we also evaluated multiple factors and their receptors in our mouse models of HALI. It was striking to note that the expression of Ang2 (MIFKO>WT>MIFTG) paralleled the mortality pattern (MIFKO>WT>MIFTG). While both Ang1 and 2 are known to act via their receptor Tie2, they have opposite effects. For example, in the context of HALI, Ang2 increases vascular permeability, while Ang1 tends to stabilize the blood vessels and decreases vascular permeability. Our previously published data [25], [34] suggest that higher levels of Ang2 in response to hyperoxia exposure in the lung are detrimental in the context of HALI. Such effects in HALI would be further compounded by decreased Ang1. In other words, an increase in Ang2, with a concomitant decrease in Ang1 would enhance HALI. In the WT mice lungs exposed to hyperoxia, Ang2 is increased, while Ang1 is decreased. In the MIFKO mice lungs exposed to hyperoxia, Ang2 is markedly increased with not much change in Ang1. The net effect of the Ang2/Ang1 expression ratio is increased Ang2, which we speculate could be contributing to enhanced HALI and increased mortality in the MIFKO mice. Since increased Ang2 has been shown in mouse models and human subjects (including premature NB with RDS) to be associated with increased mortality and/or adverse pulmonary outcomes [25], [34], [35], we speculate that a decreased concentration of Ang2 could be playing a role in the protective response of the MIFTG mice in hyperoxia.

On assessing the phenotype of the MIFKO and MIFTG mice lungs in RA, we noted a modest, but significant, increase in chord length and septal thickness, compared to WT RA controls. We observed a significant reduction in the protein expression of Ang1 and Tie2, compared to WT-RA controls. This result potentially suggests decreased Ang1 signaling, and given the importance of the Ang1-Tie2 axis in vascular integrity [36], and interaction in vascular and alveolar development [37], [38], this pathway may be responsible for the alveolar simplification phenotype in the MIFKO and MIFTG mouse lungs in RA.

MIFKO and lung MIFTG mice in RA at PN7 showed altered alveolar architecture that did not worsen further upon hyperoxia exposure (based on chord length). This reiterates two points: first, an optimal amount of MIF is required for normal lung development, and second, the severity of impaired alveolarization with lack or excess of MIF is equivalent to that caused by hyperoxia in the WT developing lung, with no additive effect of hyperoxia in the MIFKO and MIFTG mouse lung phenotype. Hyperoxia exposure in these circumstances did not exacerbate the histological changes noted in RA in the MIFKO or MIFTG mouse lungs, suggesting that there is a limiting or “plateau” effect of MIF expression–either absent or excessive, on lung development. We suggest that the need for “just the right amount” of MIF is functionally critical, and this (the “Goldilocks effect”) has been recognized by other investigators in relation to lung [39] and inner ear [40] development.

Taken together, these data suggest that restoring MIF activity within the lung to “just the right amount” is beneficial to the development of normal alveolar architecture.

VEGF-A has been noted to have a potential beneficial role in lung maturation in murine models [41], and we previously reported specifically on the role of VEGF_165_ in lung maturation using the lung-targeted, externally-regulatable TG approach [20] that was utilized in the present study. However, when we cross-bred the VEGF_165_ TG mouse onto a MIF KO background in RA, we did not find any recovery of the MIFKO pulmonary phenotype or impact on total BAL cell counts. This result was supported by our data of lack of effect on VEGF-A protein expression in the MIFKO vs. WT mice in RA. An alternative explanation could be that the increase in chord length noted in the VEGFTG mice at PN7 may have “negated” any potential recovery in lung architecture in the VEGFTG x MIFKO mouse lungs.

Another important aspect to highlight is the developmental regulation of the responses in HALI. For example, the decreased expression of Tie2 mRNA and protein, on exposure to hyperoxia, contrasts with our earlier data from adult mice [25] and could reflect the longer duration of oxygen exposure and/or developmental regulation in the NB lungs. The significant differences, and sometimes completely opposite responses in the NB vs. the adult to HALI have been noted by us [1], [3], [20], [21], and others [42], [43]. It is critical that both the developmental stage of the lung and the severity/duration of hyperoxia be taken into account when comparing studies [44]. Since the functioning of these molecules are context-dependent, studies need to be pursued in developmentally-appropriate pulmonary models.

In summary, we noted that lack of MIF is harmful, while an excess of MIF in the lung is somewhat protective in the developing lung, in terms of survival on hyperoxia exposure. However, a lack or excess of MIF in the developing lung both lead to an alveolar simplification pulmonary phenotype in RA, which was not further worsened by hyperoxia exposure in the survivors. These effects were associated with alterations in the protein expression of the Ang1, Ang2 and Tie2 angiogenic factors. We speculate that the Ang1-Ang2-Tie2 axis signaling pathway mediates the pulmonary effects of MIF in the developing lung.

We suggest that restoring or enhancing MIF activity in the developing lung, without reaching supra-physiological levels, has the potential to improve impaired alveolarization in the infants at risk for adverse outcome secondary to HALI. Additional research is needed to ascertain the ideal circumstances for augmenting MIF action in the lung, potentially via the systemic or intra-pulmonary application of small molecule MIF modulators [45] that would be protective of HALI in the developing lung.

## Supporting Information

Figure S1 *Left*: Quantitative, real-time PCR analysis of MIF mRNA in lung tissue in 6 week-old mice from the MIF-TG2 lines compared to wild-type (WT) controls. Methods and primers from [46]. *Right*: MIF protein levels in bronchoalveolar lavage fluid from mice measured by specific ELISA n = 3 mice per group. #*P*<0.01.
(DOC)
