# Supplementary material for: A Critical Regulatory Role for Macrophage Migration Inhibitory Factor in Hyperoxia-Induced Injury in the Developing Murine Lung
Source: PLoS One. 2013 Apr 29;8(4):e60560. doi: 10.1371/journal.pone.0060560 (PMC3639272; doi:10.1371/journal.pone.0060560)
Supplement: Figure S1 — Left: Quantitative, real-time PCR analysis of MIF mRNA in lung tissue in 6 week-old mice from the MIF-TG2 lines compared to wild-type (WT) controls. Methods and primers from [46]. Right: MIF protein levels in bronchoalveolar lavage fluid from mice measured by specific ELISA n = 3 mice per group. #P<0.01. (DOC) [file pone.0060560.s001.doc]

**Figure S1**

**Figure S1.** *Left:* Quantitative, real-time PCR analysis of MIF mRNA in lung tissue in 6 week-old mice from the MIF-TG2 lines compared to wild-type (WT) controls. Methods and primers from [47]. *Right:* MIF protein levels in bronchoalveolar lavage fluid from mice measured by specific ELISA n=3 mice per group. #*P*<0.01.
